# Tropisetron Suppresses Chronic Pancreatitis and Pancreatic Cancer by Blocking Interleukin 33 Expression

**DOI:** 10.3390/cancers17132087

**Published:** 2025-06-22

**Authors:** An-Na Bae, Mahsa Mortaja, YeePui Yeung, Jiao Huang, Jong Ho Park, Shadmehr Demehri

**Affiliations:** 1 Department of Anatomy, School of Medicine, Keimyung University, Daegu 42601, Republic of Korea; 2 Center for Cancer Immunology, Krantz Family Center for Cancer Research, Massachusetts General Hospital and Harvard Medical School, Boston, MA 02114, USA; 3 Cutaneous Biology Research Center, Department of Dermatology, Massachusetts General Hospital and Harvard Medical School, Boston, MA 02114, USA

**Keywords:** Interleukin 33, tropisetron, chronic inflammation, cancer prevention, chronic pancreatitis, pancreatic cancer

## Abstract

**Background/Objectives:** Chronic inflammation is a key driver of cancer. Interleukin 33 (IL-33) has emerged as a crucial factor involved in the pathogenesis of cancer-prone chronic inflammation. IL-33 functions as a cytokine and a nuclear protein to initiate chronic inflammation and cancer. However, small molecules capable of suppressing IL-33 expression to block its cytokine and nuclear functions are underexplored. **Methods:** The impact of tropisetron on IL-33 expression and its role in suppressing pancreatitis and pancreatitis-mediated pancreatic cancer were examined. **Results:** We demonstrate that tropisetron suppresses IL-33 expression, with high potential to serve as a novel therapeutic strategy for preventing chronic inflammation and its cancer sequela. Through screening 1018 Food and Drug Administration (FDA)-approved drugs, we discovered that tropisetron, a 5-hydroxytryptamine type 3 (5-HT3) antagonist commonly used to prevent and treat nausea and vomiting, effectively blocked IL-33 expression by suppressing IRF3 activation. Tropisetron inhibited pancreatitis and its progression to pancreatic cancer in mice. **Conclusions:** Tropisetron is an IL-33 inhibitor and can provide a novel therapeutic strategy to prevent and treat chronic pancreatitis and its associated cancer.

## Simple Summary

Chronic inflammation is an established risk factor for cancer development. Interleukin 33 (IL-33) cytokine has been identified as a key initiator of cancer-prone chronic inflammation. Herein, we demonstrate that tropisetron can serve as a safe and effective medication to prevent chronic inflammation and its cancer sequela. Screening a large collection of FDA-approved drugs reveals that tropisetron, which is commonly used to prevent and treat nausea, effectively suppresses IL-33 expression by reducing IRF3 phosphorylation. Tropisetron-mediated IL-33 suppression provides a novel strategy to prevent and treat pancreatic cancer and perhaps other cancers associated with chronic inflammation.

## 1. Introduction

Chronic inflammation has been identified as a key factor in cancer development, from tumor promotion and progression to metastasis [1,2]. Upon cellular damage or infection, a variety of cytokines, including IL-33, are produced and released by epithelial cells, fibroblasts, and endothelial cells [3,4]. IL-33 is a member of the IL-1 cytokine family and plays a crucial role in the onset of chronic inflammation [5,6]. IL-33 exerts its function through binding to its receptor, interleukin 1 receptor-like 1 (IL1RL1 or ST2), which activates type 2 immune responses in allergic inflammation [7,8]. This interaction stimulates T helper 2 (Th2) cells and type 2 innate lymphoid cells (ILC2s) [3,9]. In addition to its role in allergy and asthma and its involvement in the development of various chronic inflammatory diseases [7,10,11,12], IL-33 plays a crucial role in the progression of chronic inflammation to cancer [6,13].

IL-33 drives cancer progression through multiple pathways, stimulating and enhancing the growth, survival, and invasion of cancer cells and modulating the tumor microenvironment [14,15]. In addition to Th2 cells, IL-33 can activate regulatory T cells and myeloid-derived suppressor cells to promote tumor growth and evade immune surveillance [16,17]. IL-33 overexpression has been observed in several cancers, including breast cancer, colorectal cancer, lung cancer, and head and neck cancer [18,19,20,21]. This overexpression is associated with poor prognosis and reduced overall survival [16]. Thus, targeting IL-33 and its downstream signaling pathways represents a promising therapeutic approach for cancer treatment. The activation of a range of transcription factors, including nuclear factor-κB (NF-κB), interferon regulatory factor (IRF), and cAMP-response element binding protein (CREB) can lead to downstream IL-33 induction and inflammatory response [22]. Notably, IRF3 functions as a transcription factor regulating IL-33 expression in cancer-prone chronic inflammation [23,24], making it a potential target to inhibit chronic inflammation and cancer development.

Chronic pancreatitis is a significant contributor to the development of pancreatic ductal adenocarcinoma (PDAC) [25]. Patients with chronic pancreatitis exhibit a higher incidence and mortality rate of pancreatic cancer [26]. PDAC is the third leading cause of cancer-related mortality worldwide, presenting a dismal five-year relative survival rate of less than 8% [27]. PDAC is a highly aggressive malignancy with fewer than 20% of patients being candidates for surgical resection due to PDAC’s delayed clinical presentation [28]. Alternative therapeutic options such as radiotherapy and chemotherapy have yielded limited efficacy and increased toxicity [28]. Sotorasib, a KRAS G12C inhibitor, represents a recent therapeutic advancement. However, it remains insufficient for the effective treatment of PDAC [29]. PDAC is an immunologically “cold” tumor that is adept at deploying a multitude of immunosuppressive mechanisms to subvert both the innate and the adaptive immune responses [30]. Consequently, clinical trials involving immune checkpoint inhibitors (ICIs) have demonstrated limited efficacy in the treatment of PDAC [30,31].

Given the clinical challenges associated with PDAC and the limited success of current therapies, there is an urgent and unmet need for more effective preventative strategies for this cancer. Here, we identified tropisetron, which is an FDA-approved agent for the prevention and treatment of nausea and vomiting, as a potent inhibitor of IL-33 [32]. Tropisetron treatment reduced the severity of chronic pancreatitis and pancreatic cancer progression by blocking IL-33 expression. Based on these results, we propose the examination of tropisetron as a novel agent for pancreatic cancer prevention in high-risk populations.

## 2. Materials and Methods

### 2.1. Cell Lines and Transfection

Mouse keratinocyte cell line, Pam212, was obtained from Thermo Fisher Scientific company, and mouse pancreas cell line, 839WT, was generated by adapting organoids made from the pancreas of wild-type (WT) mice on the C57BL/6 background to a 2D culture system [24]. These cell lines were kept and maintained in DMEM (Thermo Fisher Scientific, Waltham, MA, USA; catalog no. 11995065) media supplemented with 10% fetal bovine serum (FBS, Thermo Fisher Scientific, catalog no. 26140079), 1X penicillin–streptomycin–glutamine (antibiotics, Thermo Fisher Scientific, catalog no. 10378016), 1X MEM non-essential amino acids solution (Thermo Fisher Scientific, catalog no. 11140050), 1X HEPES (Thermo Fisher Scientific, catalog no. 15630080), and 0.1% 2-Mercaptoethanol (Thermo Fisher Scientific, catalog no. 21985023) incubated in 37 °C and 5% CO2 environment. A total of 1.5 μg/mL poly(I:C) (Invivogen, San Diego, CA, USA; catalog no. tlrl-pic) was used for the inflammatory stimulation of Pam212 and 839WT as previously described [24]. The transfection step was conducted with Lipofectamine 2000 (Thermo Fisher Scientific; catalog no. 11668019) following the manufacturer’s recommended protocol.

### 2.2. Small Molecule Screening

FDA-approved Drug Library compounds (10 μM, Selleckchem, Houston, TX, USA, catalog no. L1300, 2019 Version) were used in the IL-33 inhibitor screening assay as previously described [24]. Each sample was measured with luminescence using an Envision 2014 plate reader (Perkin Elmer, Waltham, MA, USA; catalog no. 2014 EnVision).

### 2.3. Quantitative PCR

Mouse pancreas tissue was lysed in RLT lysis solution (QIAGEN, Hilden, Germany, catalog no. 79216) containing 0.1% MeOH using a Mini-BeadBeater-8 (BioSpec Products, Inc., Bartlesville, OK, USA). RNA extraction from the tissue or cell pellets was carried out with Trizol reagent (Thermo Fisher Scientific, catalog no. 15-596-018). The purified total RNA, obtained using the RNeasy micro kit, was quantified via a NanoDrop ND-1100 (NanoDrop Technologies, Wilmington, DE, USA). For cDNA synthesis, 1 µg of total RNA was reverse transcribed using SuperScript III Reverse Transcriptase (Thermo Fisher Scientific, catalog no. 18080085). Gene expression levels were then analyzed using a QuantStudio 3 system (Thermo Fisher Scientific) using SYBR select master mix (Thermo Fisher Scientific, catalog no. 4472908) or TaqMan Universal Master Mix II (Thermo Fisher Scientific, catalog no. 44-400-40). Primers used for the SYBR and TaqMan assays are listed in Table S1. Quantitative real-time PCR was performed in a total volume of 10 µL, comprising 4.5 µL cDNA and 5.5 µL TaqMan master mix with the relevant primers (20 µM). All assays were conducted in triplicate and normalized against *Gapdh* expression.

### 2.4. Protein Studies

Cells were lysed in RIPA buffer (Thermo Fisher Scientific, catalog no. 89900) supplemented with a protease inhibitor cocktail, EDTA-free (Thermo Fisher Scientific, catalog no. A32955). Mice tissues were homogenized and lysed in phosphate-buffered saline (PBS) containing 0.1% TWEEN-20 (Millipore Sigma, Burlington, MA, USA, catalog no. P1379). Pancreas tissues were frozen in liquid nitrogen and subsequently thawed by incubation at 37 °C prior to further processing. Lysates were sonicated for 10 s and centrifugated at 13,000 rpm. After checking the protein concentration in each sample, identical amounts of total proteins were loaded onto Mini-PROTEIN TGXTM Gels (BIO-RAD, Hercules, CA, USA; catalog no. 456-1083 and 456-1086) with Tris/Glycine/SDS buffer (BIO-RAD, catalog no. 1610732). The proteins from each sample were separated by electrophoresis (at 180 volts) and transferred to the Immobilon–P membrane (Millipore Sigma; catalog no. IPVH00010) using Transfer buffer (Boston Bioproducts, Ashland, MA, USA; catalog no. BP-190). Membranes were blocked with 3% bovine serum albumin (Thermo Fisher Scientific; catalog no. BP1600) for phospho-detected antibodies or 5% skim milk (BD Biosciences, San Jose, CA, USA; catalog no. 232100) for endogenous-detected antibodies in 1X Tris-Buffered Saline containing 0.1% TWEEN (TBS-T). After three washes with TBS-T, the membranes were exposed to appropriate primary antibodies overnight at 4 °C. The next day, the membranes were incubated with secondary antibody and developed using the Pierce ECL Western blotting substrate kit (Thermo Fisher Scientific; catalog no. 32106). Details of the antibodies used are provided in Table S1. IL-33 protein levels were measured by LEGEND MAX Mouse IL-33 ELISA kit (BioLegend, San Diego, CA, USA; catalog no. 436407) following the manufacturer’s recommended protocol. The Western blot bands were quantified using a BIO-RAD ChemiDoc Imaging System (BIO-RAD, catalog no. 17001403) and Fusion FX (Vilber, Collégien, France). Each band quantity was calculated by measuring band intensity minus the image background. Total proteins were normalized based on loading control (GAPDH) levels. Phospho-protein levels were measured as the ratio of total endogenous protein levels.

### 2.5. Animal Studies

All mice were kept in a pathogen-free environment (SPF condition) at the rodent facility of Massachusetts General Hospital in compliance with animal care guidelines. Whole-body *Il33* knockout (Il33^KO^) mice were kindly provided by Dr. Marco Colonna. Kras^LSL-G12D^ (*Kras^tm4Tyj^/J*), Tp53^flox^ (*Trp53^tm1Brnn^/J*), p48-Cre^tg^ (*Ptf11a^tm1(cre)Hnak^/RschJ*) (referred to as KPC), and WT mice on the C57BL/6 background were obtained from the Jackson Laboratory (Bar Harbor, ME, USA). Massachusetts General Hospital IACUC approved the animal studies, which were performed in the rodent facility of Massachusetts General Hospital.

### 2.6. Chronic Pancreatitis

Six-week WT mice were weighed first and then given intraperitoneal (IP) injections of 50 μg/kg caerulein (BACHEM, Torrance, CA, USA; catalog no. 4030451) in 100 μL PBS per each mouse every hour for 6 h, three times per week, over a three-week period. After three weeks, mice were sacrificed for analysis [24,33,34].

### 2.7. Caerulein-Mediated Pancreatic Cancer

KPC mice were IP injected with 50 μg/kg caerulein in 100 μL PBS every hour for 7 h, over two consecutive days, with their weight monitored during injection. All the KPC mice were sacrificed 30 days following the last caerulein injection, which is an established timeframe for pancreatic cancer development in this model [24,35,36].

### 2.8. Tropisetron Treatment

Tropisetron stock solution (5 mg/mL in DMSO) was diluted in PBS to avoid DMSO toxicity in mice. Mice were IP injected with 2 mg/kg of tropisetron in 100 μL PBS, or with an equivalent volume of a DMSO-PBS mixture (referred to as PBS) for the control group. In pancreatitis studies, the first tropisetron injection was administered one day after the first caerulein injection. In pancreatic cancer studies, the first tropisetron injection was given two days after finishing the caerulein injections. In both pancreatitis and pancreatic cancer studies, mice were treated with tropisetron every three days until they were harvested.

### 2.9. Histology and Immunofluorescence

Tissue samples were fixed overnight at 4 °C in 4% paraformaldehyde (Millipore Sigma, catalog no. P6148). Following fixation, the tissues were dehydrated using PBS and 30, 50, and 70% ethanol, processed and embedded in paraffin. Sections of 5 µm thickness from the paraffin-embedded tissues were mounted on slides, deparaffinized, and stained with hematoxylin and eosin (H&E) (Millipore Sigma, catalog no. T3260). For immunofluorescence staining, rehydrated sections were permeabilized with 0.2% Triton X-100 in 1× PBS for 5 min. Antigen retrieval was performed under high pressure using 500 µL of antigen unmasking solution (VECTOR Laboratories, Burlingame, CA, USA, catalog no. H3300) diluted in 50 mL of distilled water. The slides were washed three times with PBS containing 0.1% Tween-20. Sections were blocked with 3% bovine serum albumin and 5% goat serum for one hour at room temperature for the blocking step, then incubated overnight with primary antibodies at 4 °C. The following day, the slides were washed again and incubated with fluorochrome-conjugated secondary antibodies for two hours at room temperature. Subsequently, the sections were stained with DAPI (1:5000; Thermo Fisher Scientific, catalog no. D3571) for 3 min and washed again. Slides were mounted with Prolong Gold Antifade Reagent (Thermo Fisher Scientific, catalog no. P36930). Antibody information is provided in Table S1. Positive cells were counted in randomly selected high-power field images (200× magnification) using HALO 3.0 software (Indica Labs, Albuquerque, NM, USA).

### 2.10. Statistical Analysis

An unpaired *t*-test was employed to assess the significance of tumor-to-body weight ratios, immune cell counts, RNA and protein expression levels, and other quantitative data. Statistical differences between the three groups were evaluated using one-way ANOVA with Tukey’s multiple comparisons test. A *p*-value < 0.05 was considered statistically significant. Bar graphs represent the mean + SD.

## 3. Results

### 3.1. Tropisetron Is a Potent Inhibitor of IL-33 by Blocking IRF3 Phosphorylation

**Figure 1:**
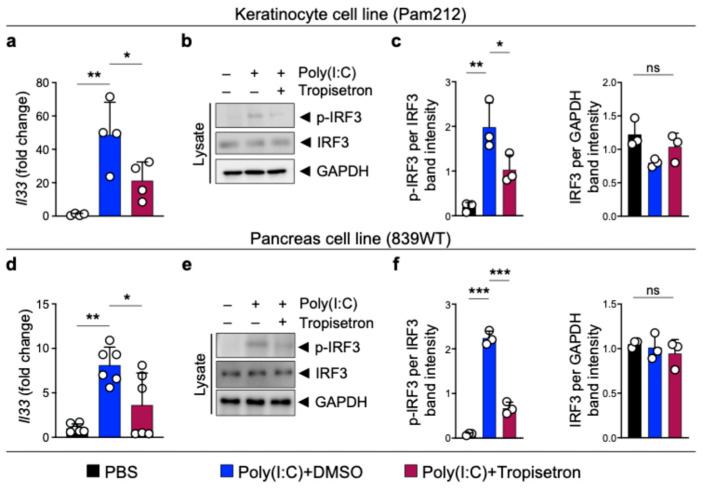
Tropisetron blocks IRF3 phosphorylation and IL-33 expression. (**a**) *Il33* expression in poly(I:C)-stimulated Pam212 cells treated with either received tropisetron or DMSO (*n* = 4 in each group). (**b**) Immunoblot of p-IRF3, IRF3, and GAPDH proteins in whole cell lysates of poly(I:C)-treated Pam212 cells that received tropisetron versus DMSO. (**c**) The ratio of p-IRF3/IRF3 and IRF3/GAPDH protein band intensity from Pam212 immunoblots (*n* = 3 in each group). (**d**) *Il33* expression in poly(I:C)-treated 839WT cells that received tropisetron or DMSO (*n* = 6 in each group). (**e**) Immunoblot of p-IRF3, IRF3, and GAPDH proteins in whole cell lysates of poly(I:C)-treated 839WT cells that received tropisetron versus DMSO. (**f**) The ratio of p-IRF3/IRF3 and IRF3/GAPDH protein band intensity from 839WT immunoblots (*n* = 3 in each group). The uncropped blots are shown in Figure S4. *: *p* < 0.05, **: *p* < 0.01, ***: *p* < 0.0001, ns: not significant, one-way ANOVA with Tukey’s multiple comparisons test. Bar graphs show mean + SD.

To suppress the cytokine and nuclear function of IL-33 in chronic inflammation, we focused on small molecules that could be safely used to block *Il33* gene expression. To identify potential small-molecule inhibitors capable of suppressing *Il33* expression, we screened 1018 FDA-approved small-molecule drugs in a luciferase-based *Il33* expression platform [24]. Among them, we identified tropisetron, a selective serotonin 5-HT3 receptor antagonist, as a highly promising candidate for IL-33 suppression based on the decreased level of *Il33*/control luminescence absorbance and RNA levels (Figure S1) [24]. To validate the effect of tropisetron on IL-33 expression, we induced IL-33 expression in the Pam212 epithelial cell line using poly(I:C), a Toll-like receptor 3 agonist [37], and treated the cells with tropisetron. Tropisetron treatment blocked *Il33* induction in Pam212 cells (Figure 1a). Furthermore, we investigated the tropisetron effect on the IRF3 signaling pathway that is known to induce IL-33 expression in chronic inflammation [23,24]. Tropisetron decreased poly(I:C)-induced IRF3 phosphorylation but not total IRF3 protein levels (Figure 1b,c). We further validated the inhibitory effect of tropisetron on *Il33* expression in the 839WT pancreas cell line (Figure 1d–f). Tropisetron also suppressed *Τnf* gene expression in poly(I:C)-treated epithelial cells, which is another downstream target of IRF3 (Figure S2) [24,38]. These findings indicate that tropisetron blocks IL-33 expression by suppressing IRF3 activation.

### 3.2. Tropisetron Alleviates Chronic Pancreatitis by Blocking IL-33

**Figure 2:**
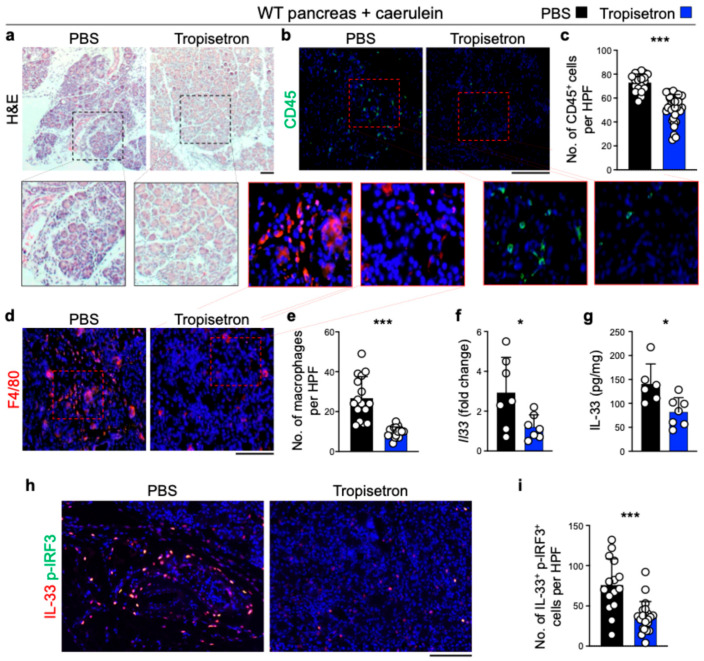
Tropisetron suppresses chronic pancreatitis in WT mice. (**a**) Representative images of hematoxylin and eosin (H&E)-stained pancreases from WT mice treated with tropisetron or PBS, following the caerulein-induced chronic pancreatitis protocol. (**b**) Representative images of CD45-stained pancreases from WT mice treated with tropisetron or PBS, following the caerulein-induced chronic pancreatitis protocol. (**c**) CD45^+^ immune cell counts in the pancreases of WT mice treated with tropisetron or PBS at the conclusion of the caerulein treatment protocol. Each dot represents cell counts from a high power field (HPF) image. Five randomly selected HPF images are included per sample (*n* = 7 in tropisetron and *n* = 4 in the PBS group). (**d**) Representative images of F4/80-stained pancreases from WT mice treated with tropisetron or PBS, following the caerulein-induced chronic pancreatitis protocol. (**e**) F4/80^+^ macrophage counts in the pancreases of WT mice treated with tropisetron or PBS at the end of the caerulein treatment protocol. Each dot represents cell counts from an HPF image. Two or three randomly selected HPF images are included per sample (*n* = 7 in tropisetron and *n* = 6 in the PBS group). (**f**) *Il33* expression in the pancreases of WT mice treated with tropisetron or PBS at the conclusion of the caerulein treatment protocol (*n* = 7 in the tropisetron group and *n* = 7 in the PBS group). (**g**) IL-33 protein levels in the pancreases of WT mice treated with tropisetron or PBS at the conclusion of the caerulein treatment protocol (*n* = 7 in the tropisetron group and *n* = 6 in the PBS group). (**h**) Representative images of IL-33 and p-IRF3-stained pancreas from WT mice treated with tropisetron or PBS, following the caerulein-induced chronic pancreatitis protocol. (**i**) IL-33 and p-IRF3 double-positive cell counts in the pancreas of WT mice treated with tropisetron (*n* = 7 mice) versus PBS (*n* = 5 mice) at the conclusion of the caerulein treatment protocol. Each dot represents cell counts from an HPF image. Three randomly selected HPF images are included per sample. Scale bars: 100 μm. *: *p* < 0.05, ***: *p* < 0.0001, two-sided unpaired *t*-test. Bar graphs show mean + SD.

**Figure 3:**
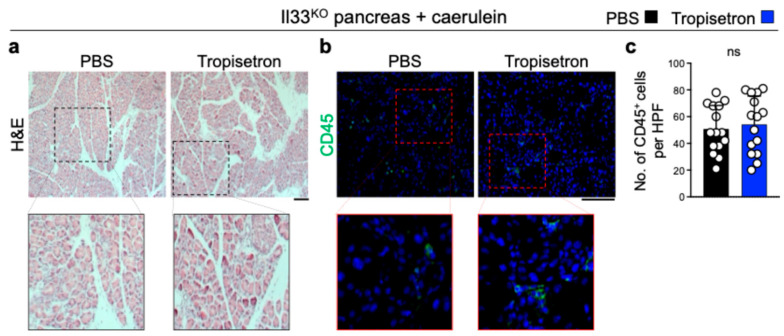
Tropisetron does not affect chronic pancreatitis in Il33^KO^ mice. (**a**,**b**) Representative images of H&E- (**a**) and CD45-stained (**b**) pancreas from tropisetron- versus PBS-treated Il33^KO^ mice at the completion of the caerulein treatment protocol. (**c**) CD45^+^ immune cell counts in the pancreases of Il33^KO^ mice treated with tropisetron or PBS at the conclusion of the caerulein treatment protocol. Each dot represents cell counts from a high-power field (HPF) image, with three randomly selected HPF images per sample. (*n* = 5 in each group). Scale bars: 100 μm. ns: not significant, two-sided unpaired *t*-test. Bar graph shows mean + SD.

To investigate the effect of tropisetron on chronic inflammation and IL-33 signaling in vivo, we induced chronic pancreatitis in WT mice by IP injection of caerulein, administered hourly for 6 h each day, three days per week, over a three-week period [24,33]. Concurrently, the mice received either tropisetron or PBS. Tropisetron treatment preserved the normal pancreas architecture compared to severe inflammation in the control pancreas (Figure 2a). Increased leukocyte and macrophage infiltrations in the pancreas are well-known markers of pancreatitis severity [24,39,40]. Tropisetron treatment significantly reduced CD45^+^ leukocytes and F4/80^+^ macrophages in the pancreas compared with the PBS-treated group (Figure 2b-e). In addition, tropisetron treatment significantly decreased *Il33* RNA, IL-33 protein levels, and double IL-33 and p-IRF3-positive cells in the caerulein-treated pancreas (Figure 2f–i). To investigate whether the therapeutic effects of tropisetron were mediated through IL-33 suppression, we subjected Il33^KO^ mice to caerulein-induced chronic pancreatitis protocol along with tropisetron versus PBS treatment. In contrast to WT mice, tropisetron treatment did not alter the severity of pancreatitis as assessed by histological evaluation and CD45^+^ leukocyte infiltration in Il33^KO^ mice (Figure 3). These findings demonstrate that tropisetron suppresses chronic pancreatitis in an IL-33-dependent manner.

### 3.3. Tropisetron Prevents Pancreatic Cancer Development in Chronic Pancreatitis by Blocking IL-33

**Figure 4:**
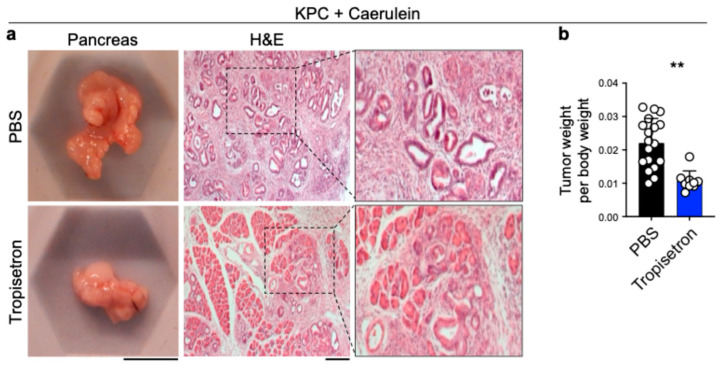
Tropisetron prevents pancreatic tumor development in KPC mice. (**a**) Representative macroscopic and H&E-stained images of the tumor-bearing pancreas from tropisetron- versus PBS-treated KPC mice that underwent caerulein-induced pancreatic cancer protocol. (**b**) The ratio of pancreatic tumor to body weight in KPC mice treated with tropisetron—versus PBS—at the completion of the caerulein-induced pancreatic cancer protocol (*n* = 9 in tropisetron and *n* = 18 in PBS group). Scale bars: 1 cm (macroscopic images) and 100 μm (histological images). **: *p* < 0.01, Two-sided unpaired *t*-test. Bar graph shows mean + SD.

To determine the effect of tropisetron on pancreatic cancer development in chronic pancreatitis, we treated KPC mice with caerulein hourly for seven hours each day over two consecutive days, followed by tropisetron or PBS treatment two times a week for four weeks [24,41]. Tropisetron-treated KPC mice exhibited a notable reduction in pancreatic tumor size compared to the control group, as evidenced by a lower tumor weight to body weight ratio (Figure 4). In contrast, tropisetron treatment did not influence the pancreatic tumor development in caerulein-treated KPC mice lacking *Il33* expression (Il33^KO^ KPC, Figure S3).

**Figure 5:**
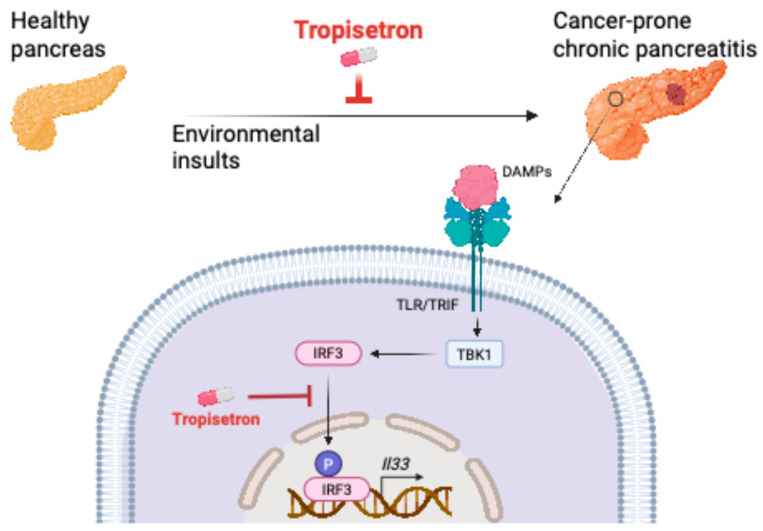
Schematic diagram of tropisetron mechanism of action in chronic pancreatitis and its cancer sequela. DAMPs: damage-associated molecular patterns. Created in BioRender. Demehri, S. (2025) https://BioRender.com/pgjpbhf (accessed on 19 June 2025).

These findings indicate that tropisetron effectively prevents chronic inflammation and its associated cancer in the pancreas by blocking IL-33 expression (Figure 5).

## 4. Discussion

Our findings highlight tropisetron’s ability to suppress chronic inflammation and cancer development in the pancreas. Tropisetron exerts this capacity by inhibiting the IRF3-IL-33 signaling pathway, which is activated by prolonged exposure to environmental insults [23,24]. The IRF3-IL-33 pathway is stimulated by the recognition of damage-associated molecular patterns that bind to TLR3/4 and initiate downstream IRF3 signaling [42].

Tropisetron effectively inhibits IL-33 expression by blocking the phosphorylation of IRF3, which helps reduce the risk of chronic pancreatitis and pancreatic cancer in mice with potential implications for humans. As an FDA-approved small molecule, tropisetron may offer a safe and accessible approach to preventing inflammation-driven cancers, particularly in high-risk individuals. This study highlights the previously unrecognized effect of tropisetron in pancreatic cancer prevention. The lower molecular weight of tropisetron may offer an advantage for tissue penetration, particularly into deep-seated organs such as the pancreas [43].

Tropisetron was originally designed to be an antagonist for the serotonin receptor [44]. Tropisetron is widely used globally to prevent chemotherapy-induced vomiting in patients [32,45]. Serotonin is known to modulate immune responses and has been associated with various inflammatory conditions, including asthma and colitis [46]. Through interactions with multiple receptor subtypes, serotonin influences cytokine secretion and immune cell activity [47]. The stimulation of the serotonin receptors has been shown to increase the secretion of pro-inflammatory cytokines, including IL-6, TNF-α, and IL-1β [48,49]. However, serotonin’s immunomodulatory effects appear to be dependent on its concentration and the underlying mechanism driving cytokine secretion remains unknown. Although the observed tropisetron’s effect on IL-33 suppression points to the potential role of serotonin in IL-33 expression, the role of the 5-HT3 receptor in IL-33 regulation is largely unknown. Future studies are warranted to investigate the role of the serotonin and 5-HT3 receptor in cancer promotion and its potential implications for novel therapeutic interventions. This understanding can open avenues for the development of innovative treatment strategies to improve current therapeutic outcomes for patients affected by chronic inflammation and cancer. However, the use of tropisetron as a therapeutic agent presents certain limitations due to the widespread expression of 5-HT3 receptor on various cell types, including immune cells, neurons, and epidermal cells [50,51,52]. Therefore, the development of a pancreas-specific drug delivery system and the use of lower doses in combination therapy may facilitate the therapeutic application of tropisetron in cancer by enhancing its tumor-suppressing capacity and minimizing off-target effects [53,54].

Pancreatic cancer exhibits low immunogenicity due to the high infiltration of immunosuppressive immune cells, such as regulatory T cells and myeloid-derived suppressor cells [55,56]. ICI therapy has shown limited success in pancreatic cancer [56,57]. Several studies have indicated that the efficacy of ICI therapy depends on the tumor microenvironment, particularly through the reactivation of T cells [58,59]. Our study demonstrates that tropisetron exerts a cancer-preventive effect against pancreatic cancer by reducing IL-33 expression in pancreatitis. However, future investigations into its effects on the tumor microenvironment of established PDAC are warranted. Previous research has identified regulatory T cells and myeloid-derived suppressor cells as primary targets of IL-33 [5,60], reinforcing the potential benefit of tropisetron-mediated IL-33 suppression in reversing the immunosuppressive tumor microenvironment in PDAC. Other research has shown the tumor-promoting role of IL-33 by activating type 2 immune response in pancreatic cancer [61]. Importantly, by blocking IRF3 phosphorylation, tropisetron likely suppresses other IRF3 target genes, including TNF-α [24]. Although IL-33 mediates the pancreatic cancer-preventive effect of tropisetron in our studies, future studies are essential to elucidate the role of tropisetron in modulating the broader immune response and tumor microenvironment. Such insights will pave the way for innovative therapeutic strategies to enhance current treatments for patients affected by pancreatic cancer.

## 5. Conclusions

Our study reveals that tropisetron, a 5-HT3 receptor antagonist, blocks IL-33 expression by inhibiting IRF3 activation and effectively reduces chronic pancreatitis and prevents pancreatic cancer. Targeting the IRF3 pathway and IL-33 expression with safe small molecules may provide innovative therapeutic strategies to tackle inflammation-driven pancreatic cancer.

## Data Availability

All data needed to evaluate the conclusions in the paper are present in the main text and/or the Supplementary Materials.

## Supplementary Materials

The following supporting information can be downloaded at https://www.mdpi.com/article/10.3390/cancers17132087/s1: Figure S1: small molecule IL-33 inhibitor discovery platform; Figure S2: Tropisetron blocks Tnf expression in epithelial cells; Figure S3: Tropisetron does not affect tumor development in Il33^KO^ KPC mice; Figure S4: Full pictures of the Western blots; Table S1: Antibodies, staining kits, and primers information.
