# Supplementary material for: Tropisetron Suppresses Chronic Pancreatitis and Pancreatic Cancer by Blocking Interleukin 33 Expression
Source: Cancers (Basel). 2025 Jun 22;17(13):2087. doi: 10.3390/cancers17132087 (PMC12248589; doi:10.3390/cancers17132087)
Supplement: Supplementary file 1 [file cancers-17-02087-s001.zip › cancers-3649519-supplementary.pdf]

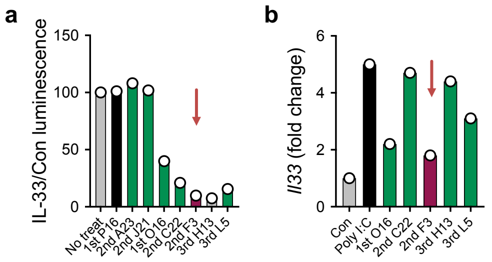

Figure S1. Small molecule IL-33 inhibitor discovery platform. (a) Candidate compounds with inhibitory effect on *Il33* reporter. **(b)** Candidate inhibitory compounds' effect on *Il33* expression induced by poly(I:C) in Pam212 cells. The red arrow points to tropisetron (labeled as F3 in the screening process).

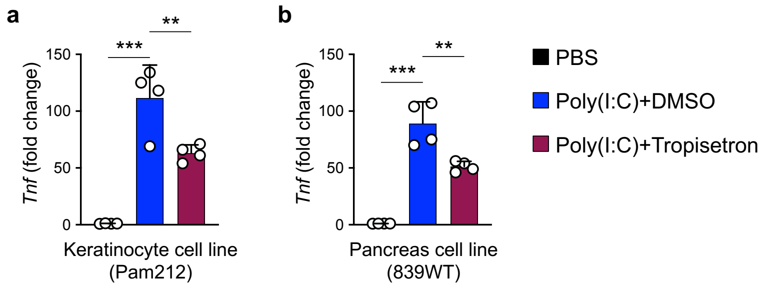

Figure S2. Tropisetron blocks Tnf expression in epithelial cells. (**a**) *Tnf* expression in poly(I:C)-treated Pam212 cells that received tropisetron versus DMSO control (n=4 in each group). (**b**) *Tnf* expression in poly(I:C)-treated 839WT pancreatic cells that received tropisetron versus DMSO (n=4 in each group). **: $p < 0.01$, ***: $p < 0.0001$, one-way ANOVA with Tukey's multiple comparisons test. Bar graphs show mean + SD,

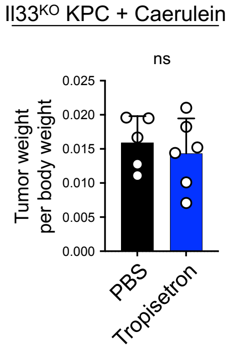

Figure S3. Tropisetron does not affect tumor development in $Il33^{KO}$ KPC mice. The ratio of pancreatic tumor per body weight in tropisetron- versus PBS-treated $Il33^{KO}$ KPC mice at the completion of caerulein-induced pancreatic cancer protocol (n=6 in tropisetron and n=5 in PBS group). ns: not significant, unpaired t-test. Bar graph shows mean + SD.

Table S1. Antibodies, staining kits, and primers information.

Western blotting Antibodies

**Primary antibodies (Clone)**	**Catalog number**	**Dilution factor**	**Company**
p-IRF3 (D601M)	29047	1:1000	Cell Signaling Technology (Danvers, MA, USA)
IRF3 (D83B9)	4302	1:1000	Cell Signaling Technology
GAPDH (D16H11)	5174	1:1000	Cell Signaling Technology
Secondary antibody			
Peroxidase Goat Anti-Rabbit IgG	115-035-003	1:5000	Jackson ImmunoResearch (West Grove, PA, USA)

Immunofluorescence staining antibodies

Primary antibodies	**Catalog number**	**Dilution factor**	**Company**
CD45 (poly-clonal)	AB10558	1:500	Abcam (Waltham, MA, USA)
F4/80 (BM8)	123101	5 ◎g/mL	Biolegend (SanDiego, CA, USA)
p-IRF3 (D601M)	29047	1:500	Cell Signaling Technology (Danvers, MA, USA)
IL-33 (#ALX804840C100)	Nessy-1	1:200	ENZO (Farmingdale, NY, USA)
Secondary antibodies			
Goat anti-Rabbit IgG, Alexa Fluor 488 conjugate	A11034	1:200	Thermo Fisher Scientific (Waltham, MA, USA)
Goat anti-mouse IgG, Alexa Fluor 568 conjugate	11004	1:200	Thermo Fisher Scientific

Primers

**qPCR primers *(Taqman)***	**Catalog number**	**Company**
Il33	Mm.PT.58. 12022572	Integrated DNA Technologies (Coralville, Iowa, USA)
Gapdh	Mm.PT.39a.1	Integrated DNA Technologies

**qPCR primers *(SYBR)***	**Forward primer**	**Reverse primer**
Tnf	CCCTCACATCAGATCATCTTCT	GCTACGACGTGGGCTACAG
Gapdh	AATGTGTCCGTCGTGATCTGA	GATGCCTGCTTCACCACTTCT
