# Supplementary figures and images for: Tropisetron Suppresses Chronic Pancreatitis and Pancreatic Cancer by Blocking Interleukin 33 Expression
Source: Cancers (Basel). 2025 Jun 22;17(13):2087. doi: 10.3390/cancers17132087 (PMC12248589; doi:10.3390/cancers17132087)

Figure 1b,c.

Main data

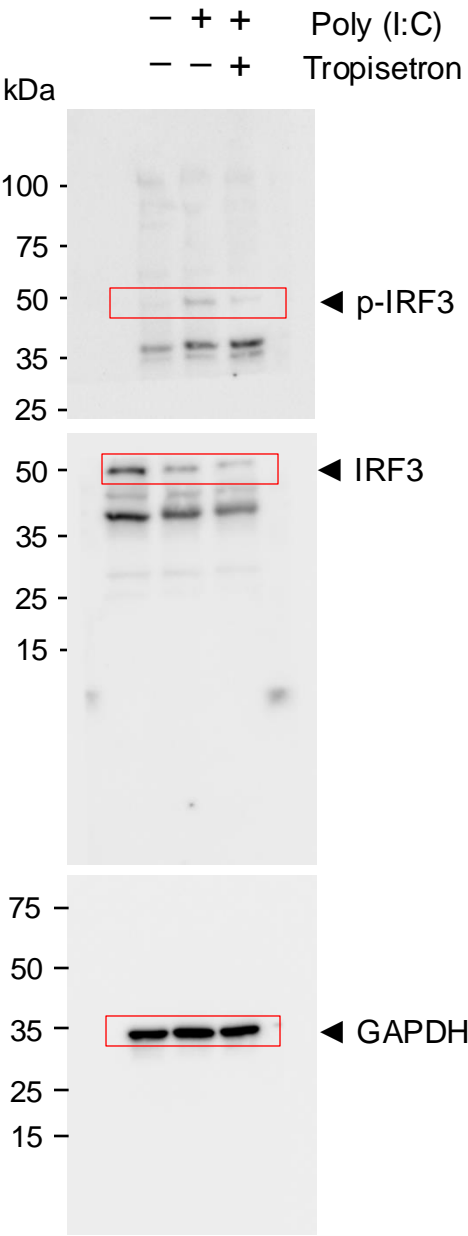

2nd test

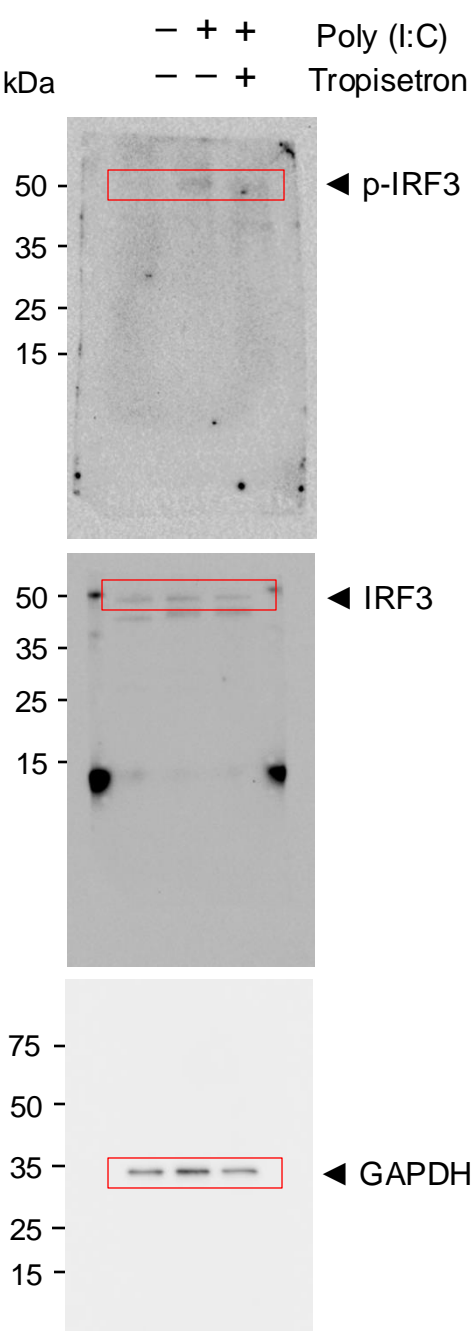

3rd test

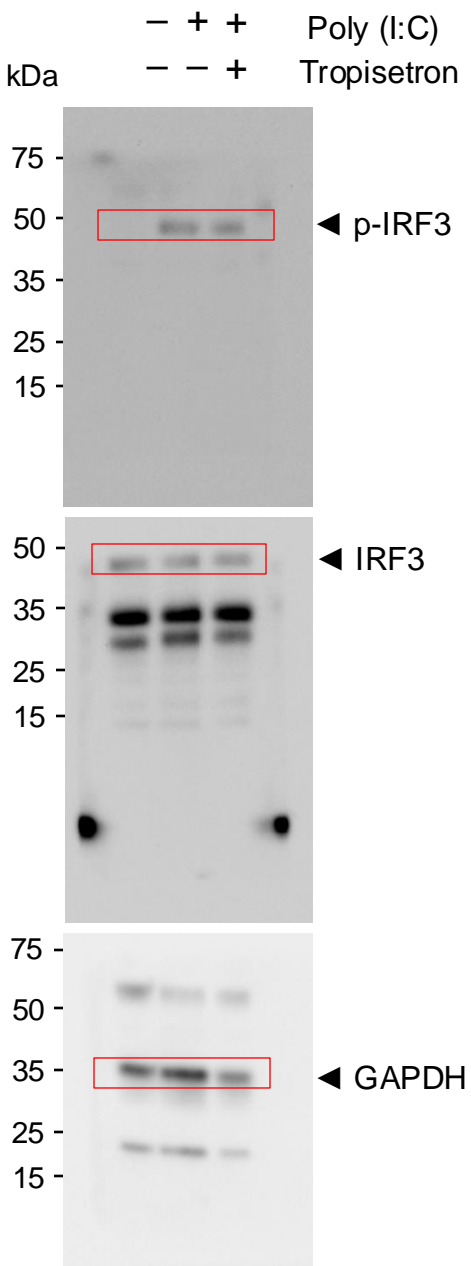

Figure 1e,f.

Main data

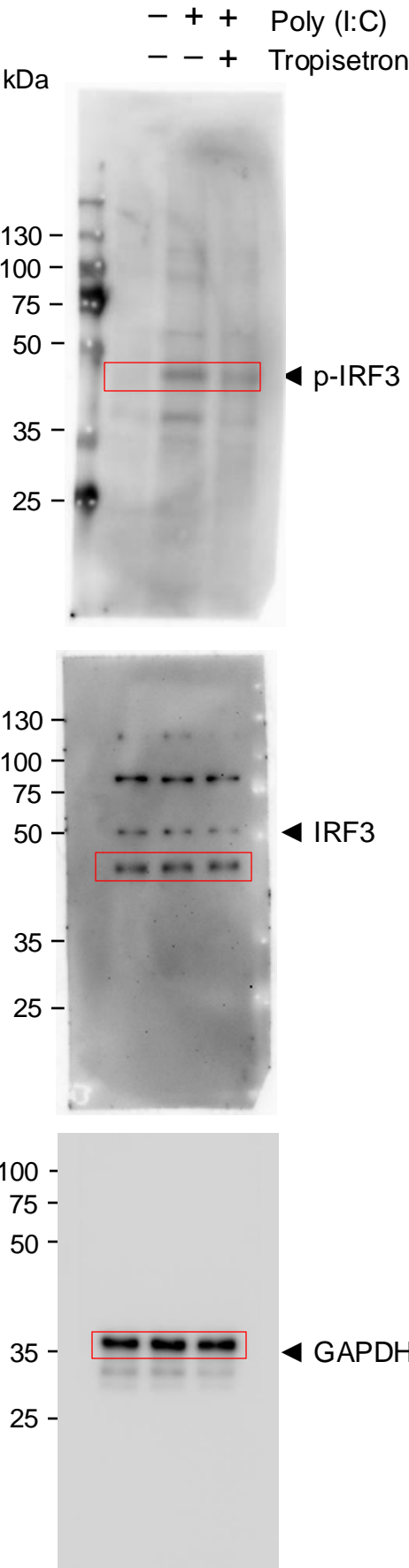

2nd test

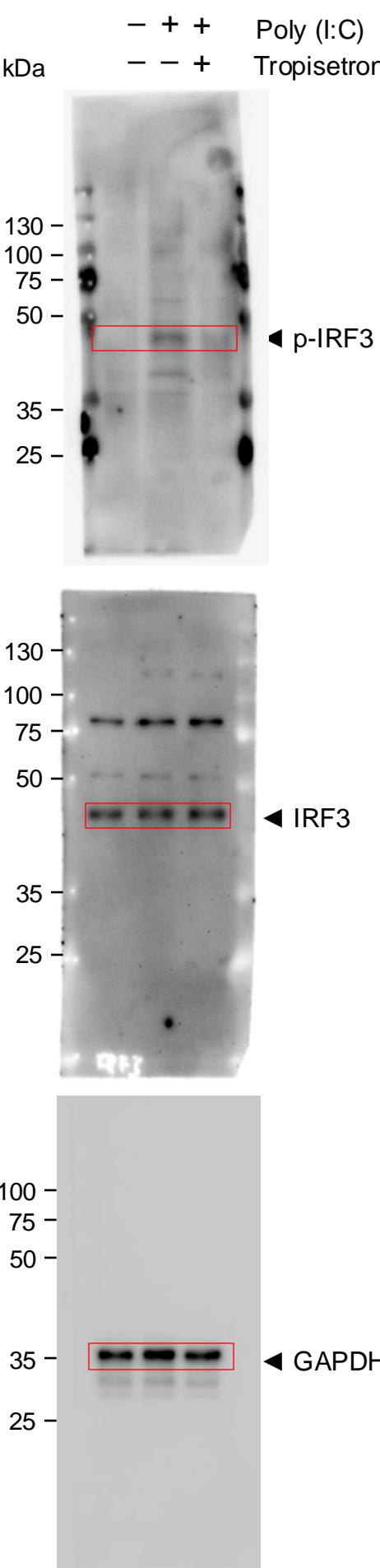

3rd test

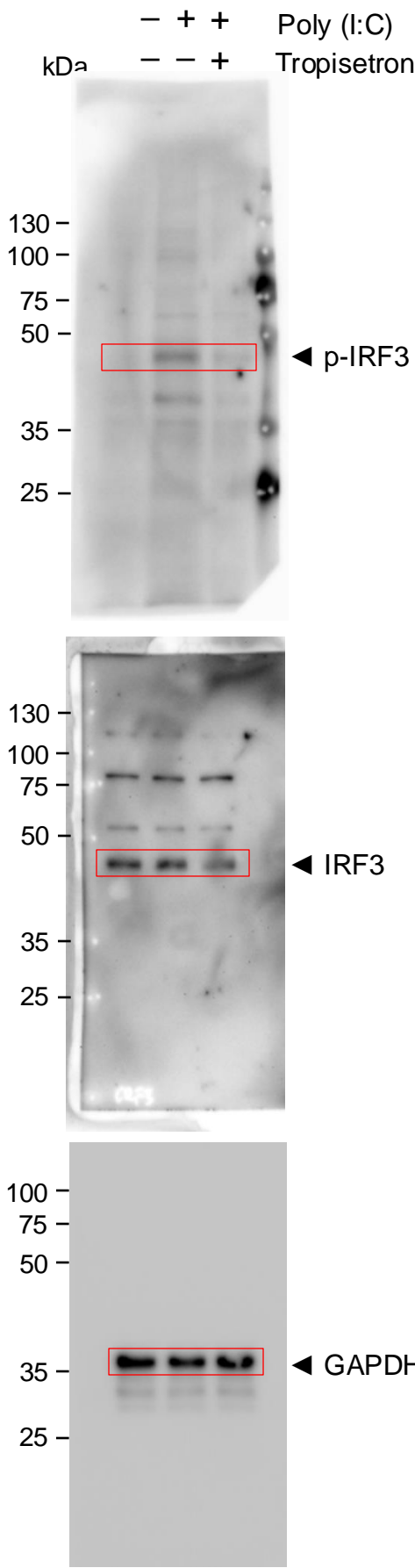

Supplement: Supplementary file 1 [file cancers-17-02087-s001.zip › cancers-3649519-Figure S4.pdf]
